# Meetings of Societies

**Published:** 1929

**Authors:** 


### Meetings of Societies.

### Bristol Medico-Chirurgical Society.

The seventh meeting of the session was held on Wednesday, 10th April, 1929, the President in the Chair.

Dr. C. Dix showed a case of ichthyosis.

Dr. H. H. Carleton read a paper on "**The Clinical Significance of Sleep Disorders.**"

Mr. J. Paul Bush, C.M.G., C.B.E., Ch.M., M.R.C.S., was elected an Honorary Member of the Society.

---

The eighth meeting of the session was held on Wednesday, 8th May, 1929, the President in the Chair.

Mr. Duncan Wood showed a case of fracture-dislocation of both astragali.

Mr. A. W. Adams demonstrated a specimen of double ureter.

Dr. F. Mayes showed skiagrams from a case of Paget's disease.

Mr. S. V. Stock read a paper on some observations on anæsthesia, which will be reported in our next issue.

Dr. Taylor read notes on "**A Case of Primary Malignant Disease of the Spleen,**" published on page 121 of this issue.

### Discussion on Dr. Taylor's Notes.

Mr. C. A. Moore spoke of the effect of radium in this condition: the size of the spleen diminished very rapidly. Adrenalin has a very transient but well-marked effect in diminishing the bulk of the spleen. In operating, the adrenalin should be injected only when everything is ready for the actual removal. He commented on the good temperamental effect seen in this patient after splenectomy.

Dr. A. D. Fraser said that he considered this a case of Gaucher's disease in a malignant form.

